# Plant-made *Salmonella* bacteriocins salmocins for control of *Salmonella* pathovars

**DOI:** 10.1038/s41598-018-22465-9

**Published:** 2018-03-06

**Authors:** Tobias Schneider, Simone Hahn-Löbmann, Anett Stephan, Steve Schulz, Anatoli Giritch, Marcel Naumann, Martin Kleinschmidt, Daniel Tusé, Yuri Gleba

**Affiliations:** 1 Nomad Bioscience GmbH, Biozentrum Halle, Weinbergweg 22 D-06120 Halle (Saale), Germany; 2 Fraunhofer Institute for Cell Therapy and Immunology, Department of Drug Design and Target Validation, Biozentrum Halle, Weinbergweg 22 D-06120 Halle (Saale), Germany; 3 DT/Consulting Group, 2695 13th Street, Sacramento, CA 95818 USA

## Abstract

*Salmonella enterica* causes an estimated 1 million illnesses in the United States each year, resulting in 19,000 hospitalizations and 380 deaths, and is one of the four major global causes of diarrhoeal diseases. No effective treatments are available to the food industry. Much attention has been given to colicins, natural non-antibiotic proteins of the bacteriocin class, to control the related pathogen *Escherichia coli*. We searched *Salmonella* genomic databases for colicin analogues and cloned and expressed in plants five such proteins, which we call salmocins. Among those, SalE1a and SalE1b were found to possess broad antimicrobial activity against all 99 major *Salmonella* pathovars. Each of the two salmocins also showed remarkably high potency (>10^6^ AU/µg recombinant protein, or >10^3^ higher than colicins) against major pathogenic target strains. Treatment of poultry meat matrices contaminated with seven key pathogenic serovars confirmed salmocin efficacy as a food safety intervention against *Salmonella*.

## Introduction

*Salmonella* is a rod-shaped Gram-negative bacterium of the *Enterobacteriaceae* family. *Salmonella enterica* is the type subspecies and is further divided into six subspecies that include over 2500 serovars. *S. enterica* infections are common and are the leading cause of gastroenteritis worldwide^1^. *Salmonella* causes an estimated 1 million illnesses in the United States each year, resulting in an estimated 19,000 hospitalizations and 380 deaths^2^ (https://www.cdc.gov/salmonella/index.html). Over the last five years (2012–2017), 51 *Salmonella* outbreaks have been recorded in USA. Most of the food poisonings were due to contaminated poultry or vegetables and fruits, but also red meats and fish (https://www.cdc.gov/salmonella/outbreaks.html). Worldwide, *Salmonella* contamination in the food chain is one of four key global causes of diarrhoeal diseases, with 550 million annual illnesses (https://www.who.int/mediacentre/factsheets/fs139/en/), and with exposure routes for non-typhoidal *Salmonella* detected in all food categories^3^.

The prevention and treatment of *Salmonella* infections, and the reduction of contamination of food and feed, have traditionally relied on generic methods such as continuous cold chains, thorough cooking, pasteurization and proper animal farm and food processing plant hygiene. The advantage of this approach is that the interventions do not require much prior knowledge about the specific *Salmonella* strains needing control. However, physicochemical interventions such as heating or treating food with oxidizing agents, acids or salts involve occupational risks and could change the properties or flavor of the treated food in undesirable ways^4^. Clinically, options for treating non-typhoidal *Salmonella* infections with existing chemistries are limited due to the potential for increased levels of excretion and the development of antibiotic resistance by *Salmonella*^5,6^. Therefore, there exists an urgent need for effective yet safe approaches for preventing *Salmonella* infections, and for compatible methods for preventing or minimizing contamination of food and feed with *Salmonella*.

In search for new alternatives to antibiotics to control pathogenic strains of the related Gram-negative bacterium *Escherichia coli*, much attention has recently been given to colicins. These are natural non-antibiotic antibacterial proteins (bacteriocins), produced by certain strains of *E. coli* (hence the name colicins) that kill or inhibit the growth of other strains of *E. coli* to establish ecological dominance^7–9^. Colicin-like bacteriocins are structurally organized in three domains: an N-terminal translocation domain responsible for transfer across the cell envelope mediated by bacterial translocator proteins, a central receptor-binding domain responsible for interaction with bacterial outer membrane receptor proteins and a C-terminal cytotoxic domain responsible for antibacterial cytotoxic activity. Depending on the translocation system employed, colicins are divided into two groups: group A (Tol system, e.g. TolABQR proteins involved in colicin import) and group B (Ton system, e.g. TonB, ExbBD proteins involved in colicin import). Based on the bactericidal activity provided by the cytotoxic domain, colicins are classified as pore-forming, nucleases, or inhibitors of peptidoglycan synthesis. To protect bacterial cells producing colicins from their cytotoxic activity, specific inhibitors called immunity proteins are simultaneously produced^7,8^. Recently, we have expressed in plants and characterized 12 colicins and found that they are excellent candidates as food additives for controlling *E. coli* in food^10^. The U.S. Food & Drug Administration (FDA) twice granted our plant-produced colicins GRAS (Generally Recognized As Safe) status as antimicrobials for application to fruits and vegetables (GRN 593, https://www.accessdata.fda.gov/scripts/fdcc/index.cfm?set=GRASNotices&id=593) and meat products (GRN 676, https://www.accessdata.fda.gov/scripts/fdcc/?set=GRASNotices&id=676), thus paving the way to commercialization of colicins as food additives or food processing aides for control of foodborne *E. coli* infections.

The two genera, *Escherichia* and *Salmonella*, are closely related, and *Salmonella* strains have been known to sometimes harbor colicin genes^11–14^; therefore, as a part of this study, we evaluated almost all (21) known colicins against major pathogenic strains of *Salmonella*, and found that Group B colicins provided moderate control, which, however, was insufficient for practical applications. Since *Salmonella*-specific colicin analogues have never been looked into, we then searched *Salmonella* genomic databases for colicin analogues and cloned and expressed five such proteins, which we called salmocins (for *Salmonella* bacteriocins). All plant-expressed salmocins showed high antibacterial activity, and included different modes of action. Two porin-type bacteriocins in particular, SalE1a and SalE1b, were each found to possess broad antimicrobial activity against all 99 major pathogenic *Salmonella* strains tested with remarkably high potency (average >10^6^ arbitrary activity units (AU)/µg protein, up to 3 orders of magnitude higher than colicins). We also evaluated salmocins as antibacterial agents on meat matrices, and compared the intra- and inter-specific activities of salmocins and colicins. Our results show for the first time the practical application of salmocins as food safety interventions as well as their potential as novel non-antibiotic bactericides.

## Results

### Green plants are efficient expression hosts for production of functional colicins and salmocins

Screening of 23 bacteriocins (colE2, colE3, colE5, colE6, colE7, colE8, colE9, colD, colIa, colIb, colN, colK, colB, colA, colR, colY, colM, col5, col10, colS4, cloacin DF13, colU, col28b), most of them *E. coli* colicins, for antimicrobial activity against *Salmonella enterica* ssp. *enterica* serovars revealed that Group A bacteriocins tested (colicins E2, E3, E5, E6, E7, E8, E9, A, N, K, R, Y, U, 28b and cloacin DF13), which utilize the Tol translocation machinery for import to susceptible bacteria, were non-effective (Supplementary Fig. 1). Conversely, activity against *Salmonella* was confirmed for colicins M, Ia, Ib, 5, 10 and S4, in agreement with literature reports. *Salmonella* serovar Typhimurium was reported to be insensitive to Group A colicins E1, E2 and E3^15,16^ and it was demonstrated that the TolQRA region of *Salmonella* serovar Typhimurium has differential levels of expression, means of regulation, and, likely, functions from its corresponding region in *E. coli*^17^, indicating that although the BtuB receptor utilized by these colicins is functional, but translocation is impaired. Sensitivity of *Salmonella* to colM was described decades ago^16^ and NCBI database searches showed that analogues of *E. coli* colicins Ia, Ib, M, and B seem to be widely distributed in *Salmonella* with 99–100% identity in amino acid sequence.

Due to relatively low overall antimicrobial activity, we concluded that the efficient control of all selected disease-relevant *S. enterica* serovars by colicins M, Ia, Ib, 5, 10 and S4 was not feasible. Therefore, putative bacteriocin genes from *Salmonella* were selected from the NCBI database on the basis of homology to the activity domains of *E. coli* colicins but with differences in amino acid composition in translocation and receptor protein domains of Group A colicins (Supplementary Fig. 2). The cut-off was set to not less than 70% identity on amino acid level for cytotoxicity domain to ensure sufficient similarity. For the translocation domain, the cut-off was set to not more than 80% identity to ensure sufficient difference. The latter rule had one exception: the translocation domain of SalE3 shared 95% identity to that of colE3 which could explain the lowest degree of anti-*Salmonella* activity of SalE3 among salmocins. Five *Salmonella* bacteriocins, which we call salmocins, representing 3 activity groups (DNase, RNase, pore-forming) of analogous proteins, were selected and designated Sal, followed by additional letters designating the highest similarity to its corresponding *E. coli* colicin.

**Figure 1:**
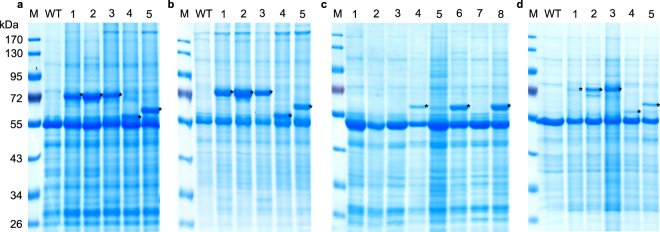
Expression of salmocins in plants. Transient expression in *N. benthamiana* upon vacuum infiltration (**a**) or syringe infiltration (**b**) with agrobacteria carrying TMV or TMV and PVX vectors. Coomassie-stained SDS protein gels loaded with samples (**a**,**b**) corresponding to 3 mg FW plant material, (**a**) crude extracts prepared with 2× Laemmli buffer or (**b**) TSP extracts prepared with 50 mM HEPES pH 7.0, 10 mM K acetate, 5 mM Mg acetate, 10% (v/v) glycerol, 0.05% (v/v) Tween 20^®^, 300 mM NaCl. (**c**) Inducible expression of salmocin SalE1b in stable transgenic *Nicotiana benthamiana* plants. Loading with crude extracts corresponds to 3 mg FW extracted with 2× Laemmli buffer from (lanes 1, 3, 5, 7) non-induced plant material or (lanes 2, 4, 6, 8) plant material 4 dp induction with ethanol. (lanes 1, 2) *N. benthamiana* WT plant, (lanes 3, 4), (lanes 5, 6), (lanes 7, 8) different transgenic plant candidates for single copy T-DNA insertion of T0 generation (#4, 12, 37 for SalE1b). (**d**) Transient expression in *Spinacia oleracea* cv. Frühes Riesenblatt upon syringe infiltration with agrobacteria carrying TMV or TMV and PVX vectors. Loading of TSP extracts corresponds to 3 mg FW plant material extracted with 5 vol. 150 mM NaCl. Plant material was harvested (**a**) 5 dpi (days post infiltration) for SalE1b, 6 dpi for SalE3, SalE7 and SalE1a or 7 dpi for SalE2 or (**b**) 4 dpi for SalE1b, 5 dpi for SalE3, SalE7 and SalE1a and 6 dpi for SalE2 or (**d**) 8 dpi for SalE2, SalE3, SalE7, SalE1a and SalE1b. (**a**,**b**,**d**) Analysed extracts were prepared from plant material expressing SalE2 (lane 1), SalE3 (lane 2), SalE7 (lane 3), SalE1a (lane 4) and SalE1b (lane 5) or from (WT) non-transfected leaf tissue. SalE2 and SalE7 were co-expressed with their respective immunity proteins. Asterisks mark recombinant proteins.

Salmocin nucleotide sequences optimized for *N. benthamiana* codon-usage were cloned into a tobacco mosaic virus (TMV)-based assembled expression vector (Table 1, Supplementary Fig. 3). To prevent potential toxicity of constructs in *E. coli* for salmocins with potential nuclease activity, an intron (from *Ricinus communis cat 1* gene) was introduced. The coding sequences of immunity proteins corresponding to nuclease-active salmocins were cloned into potato virus X (PVX)-based assembled viral vectors for co-expression (Supplementary Table 1, Supplementary Fig. 3), as described^10^. Salmocins can be expressed at very high levels in plants such as *Nicotiana benthamiana* (Fig. 1a,b) as well as in edible plant hosts such as spinach (Fig. 1d). Acidic extraction resulted in efficient recovery of soluble salmocins from plant material with the concomitant elimination of native plant proteins (Supplementary Fig. 4). The recombinant protein yields in *N. benthamiana* upon soluble extraction with neutral buffer ranged from 18–37% of total soluble protein (TSP) or 1.2–1.7 g salmocin/kg fresh weight of leaf biomass, depending on the particular protein (Table 2, Fig. 1b).

**Table 1:** List of *Salmonella* bacteriocins (salmocins) used in the study. Potential cytotoxic activities, protein molecular weight (MW) and accession numbers are presented.

No.	Salmocin	Activity	MW (Da)	GenBank Accession No.
1	SalE2	DNase	61960	KTM78572.1
2	SalE3	RNase	61710	GAS18013.1
3	SalE7	DNase	62260	KSU39545.1
4	SalE1a	Pore-forming	52812	OIN35410.1
5	SalE1b	Pore-forming	57584	OIN32443.1

**Table 2:** Yield of recombinant salmocins expressed in *Nicotiana benthamiana* plants.

No.	Bacteriocin	Harvest (dpi)	Yield (mg/g FW)*			Yield (% TSP)*		
			AV	SD	N	AV	SD	N
1	SalE2	6	1.7	0.2	3	25.0	0.0	3
2	SalE3	5	1.6	0.2	3	37.0	10.4	3
3	SalE7	5	1.4	0.3	3	18.0	6.9	3
4	SalE1a	5	1.2	0.2	3	20.3	3.1	3
5	SalE1b	4	1.2	0.1	3	25.7	3.1	3

*The yield was calculated in mg/g fresh weight of plant biomass and as a percentage of TSP (based on extraction with 50 mM HEPES pH 7.0, 10 mM K acetate, 5 mM Mg acetate, 10% (v/v) glycerol, 0.05% (v/v) Tween-20, 300 mM NaCl and syringe inoculation of plant material with 1:100 dilutions of agrobacterial cultures (OD_600_ = 1.3)) and is represented as an average value and standard deviation (AV, SD) of several experiments. N, number of independent experiments. In each experiment, plant material pooled from 3 plants was analyzed for each protein.

### Production of salmocins in stable transgenic hosts

Stable transgenic *Nicotiana benthamiana* plants containing the genomic insertion of TMV-based viral vector double-inducible with ethanol for SalE1b expression (Supplementary Fig. 5) exhibited normal growth and development, and selected transgenic lines accumulated salmocins upon induction with ethanol to the expected levels (Fig. 1c).

### Salmocins SalE1a and SalE1b are the most broadly and highly active salmocins for control of *S. enterica*

**Figure 2:**
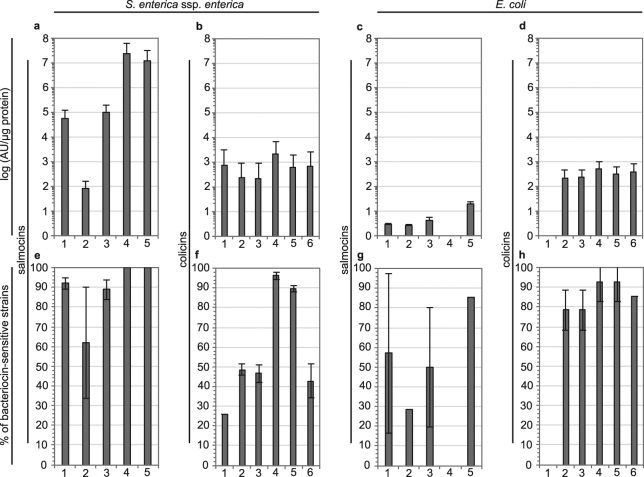
Activity spectrum of bacteriocins from *Salmonella* and *E. coli* against *Salmonella enterica* ssp. *enterica* and *E. coli* Big 7 STEC serotypes. Semi-quantitative evaluation of the specific antimicrobial activity by radial diffusion assay via spot-on-lawn-method of (**a**,**c**,**e**,**g**) salmocin- and (**b**,**d**,**f**,**h**) colicin-containing plant extracts against 36 (**a**,**e**) or 35 (**b**,**f**) *S. enterica* ssp. *enterica* strains listed in Supplementary Table 2 or 7 *E. coli* Big 7 STEC strains (**c**,**d**,**g**,**h**) listed in Supplementary Table 4. Average and standard deviation of N = 3 and N = 2 independent experiments is given in (**a**,**b**,**e**,**f**) and (**c**,**d**,**g**,**h**), respectively, for the percentage of bacteriocin-sensitive strains (**e**,**f**,**g**,**h**) and for the specific bacteriocin activity calculated in arbitrary units (AU) per µg of recombinant protein (**a**,**b**,**c**,**d**) on all tested strains. 1 – SalE2 (**a**,**c**,**e**,**g**) or colS4 (**b**,**d**,**f**,**h**); 2 – SalE3 (**a**,**c**,**e**,**g**) or col5 (**b**,**d**,**f**,**h**); 3 – SalE7 (**a**,**c**,**e**,**g**) or col10 (**b**,**d**,**f**,**h**); 4 – SalE1a (**a**,**c**,**e**,**g**) or colIa (**b**,**d**,**f**,**h**); 5 – SalE1b (**a**,**c**,**e**,**g**) or colIb (**b**,**d**,**f**, **h**); 6 – colM (**b**,**d**,**f**,**h**).

To determine the salmocin antimicrobial activity spectrum, 109 strains representing 105 *S. enterica* ssp. *enterica* serotypes were selected and screened (Supplementary Table 2). The screen included one strain each of all serotypes (except serotypes Typhi and I4,5:12:r:-) that are documented at the U.S. Centers for Disease Control and Prevention (CDC) (https://www.cdc.gov/nationalsurveillance/pdfs/salmonella-annual-report-2013-508c.pdf) as having caused at least 100 incidences of human *Salmonella* infection from 2003–2012, two strains of serotypes Typhimurium, Enteritidis and Javiana and 6 serotypes causing less than 100 incidences or not reported to CDC. In order to estimate the breadth of the activity spectrum, all strains were tested at least once and 36 or 35 strains were subsequently re-screened in triplicate experiments with salmocins and colicins, respectively (Fig. 2). The broadest antimicrobial activity spectrum was identified for salmocins SalE1a and SalE1b, which showed positive antibacterial activity against 100% and 99% of all strains evaluated, respectively. Significant breadth of activity was also observed for salmocins SalE2 (94%), SalE3 (70%) and SalE7 (95%) as reflected by their activity on the subset of 36 strains represented in Fig. 2e.

**Figure 3:**
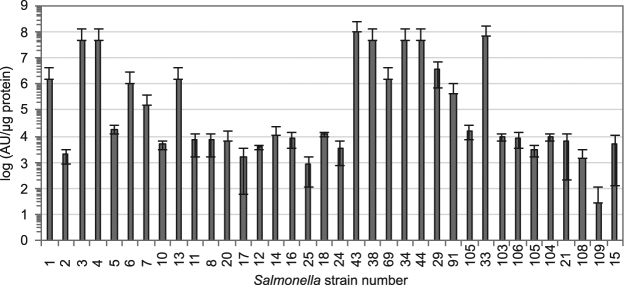
Specific activity of salmocin SalE1b against *Salmonella enterica* ssp. *enterica* serovars. Semi-quantitative evaluation of the average specific antimicrobial activity of salmocin SalE1b-containing plant total soluble extracts against 36 *S. enterica* ssp. *enterica* strains listed in Supplementary Table 2. The antimicrobial activity was tested using radial diffusion assay via spot-on-lawn-method is given as average arbitrary units (AU) per µg recombinant protein (error bars correspond to standard deviation, N = 3).

The five salmocins analysed were divided into four groups based on their ability to control major pathogenic *Salmonella* strains. Salmocins SalE1a and SalE1b were universally active, each being able to kill all tested pathovars and showing the highest average activity of higher than 10^5^ AU/µg recombinant protein on all tested strains (Fig. 2a) and in most cases higher than 10^3^ AU/µg protein against individual strains (Fig. 3, Supplementary Fig. 9). The remaining salmocins fell into two groups, with salmocins SalE2 and SalE7 in one group having a 100-fold lower average activity (<10^5^ AU/µg protein, Fig. 2a, Supplementary Figs 6, 8), and SalE3 in another group showing substantially lower average activity (10^2^ AU/µg, Fig. 2a, Supplementary Fig. 7).

In contrast to the high potencies of salmocins in inhibiting enteropathogenic *S. enterica* strains, the specific activities of colicins Ia, Ib, M, 5, 10 and S4 (Supplementary Table 3) were 2–4 orders of magnitude lower (2–3 logs AU/µg, Fig. 2b), although most of the 109 strains were inhibited by colicins Ia (92%) and Ib (90%) and about one third of strains by colicins S4 (45%), 5 (25%), 10 (29%) and M (34%), as also reflected in the susceptibility pattern of the subset of 35 strains (Fig. 2f). In general, salmocins demonstrated higher and broader activity against *Salmonella* than *E. coli* colicins. Conversely, salmocins showed low (below 10^2^ AU/µg) inter-specific and narrrow activity against *E. coli* STEC (Supplementary Table 4) strains (Fig. 2c,g, Supplementary Fig. 10).

### Salmocin SalE1a controls *Salmonella* on contaminated chicken meat matrices

**Figure 4:**
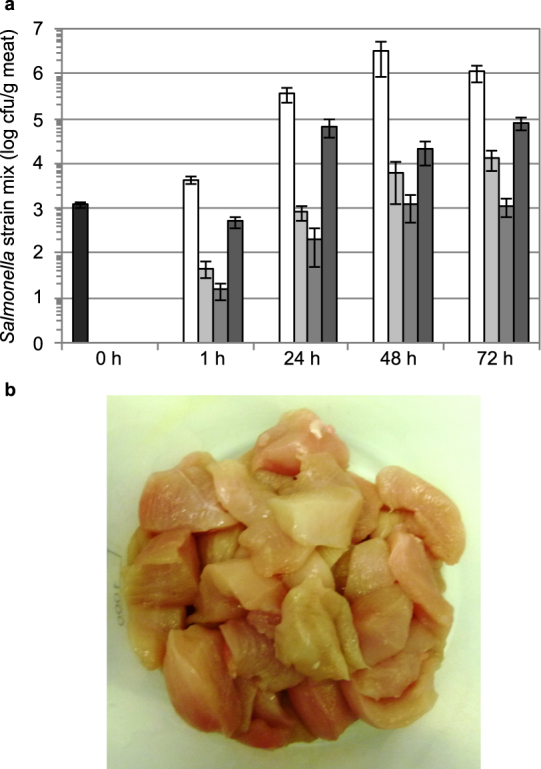
Reduction of a *S. enterica* ssp. *enterica* contamination on fresh chicken breast fillet by salmocins. (**a**) Bacterial populations recovered from meat upon storage for various periods of time at 10 °C upon salmocin treatment (black bars, initial contamination level; white bars, carrier treatment; light grey bars, bacteriocin treatment SalE1a in concentration of 3 mg/kg meat; grey bars, bacteriocin treatment SalE1a + SalE1b + SalE2 + SalE7 in concentration of 3 + 1 + 1 + 1 mg/kg meat; dark grey bars, bacteriocin treatment SalE1a + SalE1b + SalE2 + SalE7 in concentration of 0.3 + 0.1 + 0.1 + 0.1 mg/kg meat) of contaminated meat by spray-application. Error bars indicate standard deviation of biological replicates, N = 4. Statistically significant reductions in bacterial contamination were found by assessment of viable bacterial counts obtained from salmocin-treated (individual SalE1a at an application rate of 3 mg/kg meat (p-values for comparison at 1 h, 24 h, 48 h and 72 h post treatment were <0.0001, 0.0012, 0.0137 and 0.0037, respectively) or salmocin blend consisting of SalE1a + SalE1b + SalE2 + SalE7 at an application rate of 3 + 1 + 1 + 1 mg/kg meat (p-values for comparison at 1 h, 24 h, 48 h and 72 h post treatment were <0.0001, 0.0012, 0.0136 and 0.0035, respectively) and salmocin blend consisting of SalE1a + SalE1b + SalE2 + SalE7 at an application rate of 0.3 + 0.1 + 0.1 + 0.1 mg/kg meat (p-values for comparison at 1 h, 24 h, 48 h and 72 h post treatment were 0.0002, 0.004, 0.0139 and 0.005, respectively) respectively), all in relation to carrier-treated meat samples showing efficacy of salmocin treatment. (**b**) Chicken breast trims used in (**a**).

The bactericidal efficacy of plant-produced individual salmocin SalE1a as well as salmocin blends for control of *Salmonella*-contaminated meat surfaces was modeled in a simulation study. Efficacy of salmocin treatment was assessed for the extent of reduction in the pathogenic bacterial population level on salmocin-treated (individual SalE1a at an application rate of 3 mg/kg meat and salmocin blend consisting of SalE1a + SalE1b + SalE2 + SalE7 applied at 3 + 1 + 1 + 1 mg/kg meat, respectively), both in relation to plant extract control-treated, meat samples and statistically significant net reductions in viable counts of 2–3 logs CFU/g meat at all timepoints analysed were found (Fig. 4). The highest level of reduction of bacterial populations was observed for the 4-salmocin blend (concentration of 3 + 1 + 1 + 1 mg/kg meat) with up to 3.39 mean log reduction vs. carrier treatment upon 48 h of storage, which corresponds to a 99.6 mean percent reduction of bacteria. A single salmocin, SalE1a (applied at 3 mg/kg meat), was able to control *Salmonella* contamination on meat with similar efficacy to the blend of four salmocins applied at double the concentration (6 mg/kg meat total salmocin). Even a treatment with salmocins at very low dose (total salmocin 0.6 mg/kg meat; 0.3 + 0.1 + 0.1 + 0.1 mg/kg meat for a blend of SalE1a + SalE1b + SalE2 + SalE7) produced statistically significant reductions of bacterial populations of about 1 log CFU for up to 48 h of storage. Upon initial reduction of bacterial contamination, re-growth of viable bacteria was observed after 72 h, indicating that salmocins act quickly but have no prolonged technical effect on food.

### Plant-produced salmocins are digested by gastric and intestinal proteases

Although salmocins applied to raw food will be degraded during cooking, it is important to show that ingested salmocins not degraded by food processing will be degraded in the human gastrointestinal tract to prevent influencing the indigenous GI tract microflora, as well as to avoid the potential development of allergic reactions. A database search of all salmocin amino acid sequences (http://www.Allergenonline.org) revealed distal relationships but no exact matches to known allergens. To verify the low risk of allergenicity and residual activity upon ingestion, the digestibility of salmocin proteins was analysed. A complete loss or negligible residual antimicrobial activity was observed for SalE1b and SalE7 or SalE1a, respectively (Supplementary Fig. 11a). For SalE1b and SalE7, the full-length proteins disappeared upon 1 h incubation at gastric conditions. Full-length SalE1a protein was found in negligible amount after only 20 min of gastric digestion and was completely degraded upon additional incubation at duodenal conditions for 5 min. (Supplementary Fig. 11b).

### Recombinant salmocins are correctly expressed by plants

The primary structure including post-translational modifications of the plant-expressed recombinant salmocins contained in plant TSP extracts was analysed by mass spectrometry-based sequencing. Search results of each MS/MS dataset from proteolytic peptides of salmocins against the UniProt/SwissProt database confirmed the identity of each of the analysed salmocins (Supplementary Table 5). The integrity of purified salmocins SalE1a, SalE1b and SalE7 was further analysed by MS-based sequencing of protein termini using ISD and molecular mass determination methods, which confirmed that all salmocin proteins were intact upon plant expression. Post-translational modifications observed were restricted to cleavage of N-terminal methionine in case of SalE2, SalE7, SalE1a and SalE1b and N-terminal acetylation for SalE7 and SalE1a (Supplementary Table 5).

## Discussion

Perhaps the most significant outcome of our study is proof that *Salmonella* harbors genes for its own active species-specific colicin-like bacteriocins, much like other Gram-negative bacteria including *Escherichia coli*^10^ and *Pseudomonas aeruginosa*^18–20^, in addition to exploiting *E. coli* colicins. The two most interesting salmocins SalE1a and SalE1b are 69% identical at the amino acid level with most differences found in the protein region responsible for receptor binding. It is expected that both proteins use the same translocation machinery but different receptors for entry into susceptible bacterial cells. This is supported by the finding that one of the *Salmonella* strains (serotype Kintambo) was killed by SalE1a but not by SalE1b, and that for SalE1a no activity was detected against any of the *E. coli* strains tested whereas SalE1b showed the highest antibacterial effect of all the salmocins tested against *E. coli*. By salmocin SalE3, only about 60% of tested strains were inhibited at low average activity which could be explained by SalE3 having the highest identity to Group A colicins of the protein region responsible for translocation.

The other significant conclusion of this study is that plant-expressed salmocins are excellent candidate biocontrol agents for pathogenic *Salmonella*. To our knowledge, this work is the first study devoted to the novel recombinant bacteriocins, salmocins, and has demonstrated that salmocins, applied singly or as mixtures, efficiently control all major pathogenic serotypes of *Salmonella enterica* ssp. *enterica* not only *in vitro* but also in simulation studies involving contaminated meat matrices. This work clearly demonstrates the specificity, potency, and efficacy of salmocins against major enteropathogenic serotypes of *Salmonella enterica* ssp. *enterica*.

In contrast to the seven major *E. coli* pathotypes known as the “Big 7” classified by the Food Safety and Inspection Service (FSIS) branch of the U.S. Department of Agriculture (USDA) as adulterants on meat based on historical analysis of *E. coli* food poisonings (https://www.gpo.gov/fdsys/pkg/FR-2011-09-20/pdf/2011-24043.pdf), a list of major foodborne *Salmonella* strains requiring control has not been fully defined by regulatory agencies, primarily due to higher diversity of the pathovars responsible for the outbreaks. Lacking further guidance, we included in our tests 99 of 101 *S. enterica* ssp. *enterica* serotypes known to have caused at least 100 incidences of human *Salmonella* infections reported to CDC during 2003–2012 (https://www.cdc.gov/nationalsurveillance/pdfs/salmonella-annual-report-2013-508c.pdf). This number is 15 times higher than the number of *E. coli* pathovars defined by FDA (seven).

Our finding that two salmocins, SalE1a and SalE1b, each possessed broad antimicrobial activity against all 99 major pathogenic *Salmonella* strains tested as well as remarkably high activity (average > 10^6^ AU/µg), is unexpected. For comparison, colicins, which are salmocin analogues produced by *E. coli* cells, exhibit much narrower selectivity against seven *E. coli* pathovars, and mixtures of three to five colicins had to be used to efficiently inhibit strains of all “Big 7” *E. coli* STEC serotypes. Colicins also demonstrated much lower average activity against *E. coli* “Big 7” STEC strains (average < 10^3^ AU/µg), although higher activity has been observed on a strain of serotype O104:H4 (>10^5^ AU/µg)^10^ that caused major outbreaks in 2011 in Europe, and a common laboratory strain *E. coli* DH10B (>10^5^ AU/µg). Similar limited intra-specific activity and efficacy have been observed also with *Pseudomonas* pyocins^17^.

Our analysis of cross-specific activity of salmocins and colicins on *E. coli* and *Salmonella*, respectively, confirmed the expected low activity against bacteria of different genera. In particular, the activity of salmocins against “Big 7” STEC strains was negligible (less than 10^2^ AU/µg) although some salmocins (such as SalE2, SalE7 and SalE1b, but not SalE1a) were found active against *E. coli* O104:H4 (10^3^AU/µg) and laboratory strain DH10B (10^5^AU/µg) (data not shown). Similarly, the activity of colicins on *Salmonella* pathovars was found to be low, with colicins Ia and Ib being active on over 80% of strains, but with average activity of only colIa being higher than 3 × 10^3^ AU/µg (or three to four orders of magnitude less than salmocins SalE1a and SalE1b). The practical conclusion of this study is that, to combat pathotypes of both genera simultaneously, or with a single intervention, mixtures of colicins and salmocins will be required.

The salmocins SalE1a and SalE1b have been tested at different concentrations and used to simulate the effect of salmocin treatment on poultry meat matrices spiked with *Salmonella* and stored at 10 °C, providing additional evidence for the practical importance of these two salmocins as intervention agents for food safety. The issue of performance at low temperature is important because much of the food is processed and distributed using cold chains, and any new candidate antimicrobial needs to be active in applications that are compatible with the existing industrial infrastructure and process parameters.

Our data demonstrate that salmocins can be expressed at very high levels in the plant *Nicotiana benthamiana*, a standard manufacturing host for multiple biopharmaceuticals currently undergoing clinical trials^21–25^, as well as in edible plant hosts such as spinach, and that the antibacterial proteins expressed in either host are fully active. The expression levels in most cases reached 20–37% of total soluble protein or 1.2–1.7 g product/kg of fresh leaf biomass without process optimization. These findings show that salmocins are not toxic to plants, and suggest that future optimization of industrial procedures for transfection or induction in transgenic hosts and downstream recovery could be developed that are both economical and scalable to meet market demand. In sharp contrast, attempts to express bacteriocin proteins in bacterial production systems usually met with generalized host toxicity even in species other than the bacteriocins’ homologous bacterial hosts (e.g.^26,27^)_._ Thus, plants are excellent hosts for manufacturing not only phage endolysins^28,29^, but also multiple bacteriocins including colicins^10^, pyocins^20^, and now also salmocins.

Salmocins are natural non-antibiotic antibacterial proteins that offer multiple potential applications including treatment of food to eliminate pathogenic *Salmonella*, as well as use as human and animal therapeutic alternatives to antibiotics. The use of salmocins as food additives or food processing aids is especially attractive because of the magnitude of current food safety issues worldwide, and because these product candidates can be approved relatively quickly using the GRAS regulatory pathway in the USA.

## Methods

### Bacterial strains and growth conditions

*Escherichia coli* DH10B, *Salmonella enterica* ssp. *enterica* (Supplementary Table 2) and STEC (Supplementary Table 4) cells were cultivated at 37 °C in LB medium (lysogeny broth^30^). *Agrobacterium tumefaciens* ICF320^31^ cells were cultivated at 28 °C in LBS medium (modified LB medium containing 1% soya peptone (Duchefa)).

### Plasmid constructs

The methods used to construct and apply TMV- and PVX- based magnICON^®^ vectors and TMV-based vectors for EtOH-inducible expression (Supplementary Figs 3,5) were as described previously^10^.

Coding sequences of genes of interest (salmocins (Table 1) and salmocin immunity proteins (Supplementary Table 1)) were codon-optimized for *Nicotiana benthamiana*, synthesized by Thermo Fisher Scientific and were cloned into the BsaI sites of the respective destination vectors. To avoid toxicity to bacteria, for some salmocin sequences an intron of *Ricinus communis* *cat1* gene was introduced for gene synthesis (Supplementary Fig. 3), correct intron splicing was verified as described^10^.

### Plant material and inoculations

*Nicotiana benthamiana* and *Spinacia oleracea* cv. Frühes Riesenblatt plants were grown in the greenhouse (day and night temperatures of 19–23 °C and 17–20 °C, respectively, with 12 h light and 35–70% humidity). Six-week-old plants were used for inoculations. Plant transfection was done as described^10^.

### Stable plant transformation and regeneration and ethanol-induction of transgene expression

*N. benthamiana* was transformed by *Agrobacterium*-mediated leaf disk transformation using vectors for EtOH-inducible transgene expression and induction of detached leaves of T0 generation transgenic plants for salmocin expression was done as described^10^.

### Protein analysis

Plant leaf material was ground in liquid nitrogen and protein extracts were either prepared with 5 vol. 2× Laemmli buffer (crude extracts) or different buffers, e.g. 50 mM HEPES pH 7.0, 10 mM K acetate, 5 mM Mg acetate, 10% (v/v) glycerol, 0.05% (v/v) Tween-20, 300 mM NaCl (total soluble protein (TSP) extracts). The protein concentration of TSP extracts was determined by Bradford or BCA assay using Bio-Rad Protein Assay (Bio-Rad Laboratories) or Pierce™ BCA Protein Assay Kit (Thermo Fisher Scientific) and BSA (Sigma-Aldrich) as a standard. For analysis by SDS-PAGE and Coomassie-staining using PageBlue™ Protein Staining Solution (Thermo Fisher Scientific), protein extracts were denatured at 95 °C for 5 min before loading. The estimation of the percentage of recombinant colicins of TSP was done by comparison of TSP extracts to known amounts of BSA standard (Sigma-Aldrich) on Coomassie-stained SDS-PAA gels.

### Salmocin purification

Plant TSP extracts were prepared by supplementation of leaf material ground in liquid nitrogen with 5 vol. pre-chilled extraction buffers as 20 mM citric acid pH 4, 20 mM NaH_2_PO_4_, 30 mM NaCl, 0.05% Tween-80 for SalE1a and SalE7, or 20 mM citric acid pH 5.5, 20 mM NaH_2_PO_4_, 30 mM NaCl, 0.05% Tween-80 for SalE1b. Homogenates were incubated for 15 min on ice. Extracts were clarified by centrifugation for 15 min at 3515 × g and filtration using Miracloth. For SalE7 and SalE1a, clarified extracts were supplemented with 10 mg/ml diatomaceous earth. All extracts were incubated for 30 min at room temperature with constant agitation and were clarified again by centrifugation for 15 min at 3515 × g and filtered through filter discs of 8–12 µm pore size before loading for column-purification by cation exchange chromatography (CIEX) using SP-SepharoseFF for SalE1a and SalE1b and by CIEX using CM-Sepharose for SalE7.

SalE1a was step-eluted with 20 mM citric acid pH 4, 20 mM Na_2_HPO_4_, 1 M NaCl, 0.05% Tween-80 upon column wash with extraction and 45% elution buffer. Buffer exchange of SalE1a eluate was performed using U-tube concentrators (Sartorius) at molecular cut off of 10 kDa with 20 mM Na_2_HPO_4_, 10 mM citric acid, 50 mM NaCl, pH 6. SalE1b was step-eluted with 10 mM citric acid pH 7.5, 20 mM Na_2_HPO_4_, 100 mM NaCl upon column-wash with extraction buffer and 8% elution buffer. SalE7 was step-eluted using 50 mM Na_2_HPO_4_, 10 mM citric acid, 50 mM NaCl upon column-wash with extraction buffer and 50% elution buffer. Eluted fractions of SalE1b and SalE7 or of SalE1a upon buffer exchange were snap-frozen in liquid nitrogen and freeze-dried.

### Salmocin antimicrobial activity determinations

Semi-quantitative determination of salmocin antimicrobial activity was done by spot-on-lawn/radial diffusion assay on serial dilutions of plant TSP extracts containing salmocins as employed in Schulz *et al*.^10^. Salmocin specific antimicrobial activity was calculated in arbitrary activity units (AU) per µg recombinant protein using the reciprocal of the highest dilution with visible growth reduction effect on bacterial cells and the recombinant protein content of solutions analysed.

### Reduction of bacterial populations on food

Chicken breast fillet was purchased from a local supermarket. Nalidixic acid resistant mutants of strains of *S. enterica* ssp. *enterica* serovars Enteritidis (strain ATCC^®^13076™*), Typhimurium (strain ATCC^®^14028™*), Newport (strain ATCC^®^6962™*), Javiana (strain ATCC^®^10721™*), Heidelberg (strain ATCC^®^8326™*), Infantis (strain ATCC^®^BAA-1675™*) and Muenchen (strain ATCC^®^8388™*) were individually grown in LB medium supplemented with 25 µg/ml nalidixic acid to stationary phase, diluted with fresh LB and grown to exponential phase. For contamination of poultry, bacterial cultures were diluted with LB medium to OD_600_ = 0.001 (~2 × 10^5^ cfu/ml) and mixed 1:1:1:1:1:1:1. A pool of chicken breast fillets cut into pieces of about 20 g weight was inoculated with 1 ml of a mixture of 7 *S. enterica* strains at ~2 × 10^5^ CFU/ml density per 100 g of meat at room temperature resulting in an initial contamination level of meat matrices of about 3 log CFU/g of a 7-serotype mixture of pathogenic *S. enterica*; attachment of bacteria to meat surfaces was allowed for 30 min at room temperature. Subsequently, chicken breast trims were treated by spraying (10 ml/kg) with either plant extract control (TSP extract of WT *N. benthamiana* plant material with no salmocins, prepared with 50 mM HEPES pH 7.0, 10 mM K acetate, 5 mM Mg acetate, 10% (v/v) glycerol, 0.05% (v/v) Tween-20, 300 mM NaCl), or salmocin solutions (either individual or mixtures of TSP extracts of *N. benthamiana* plant material expressing salmocins SalE1a, SalE1b, SalE2 and SalE7 prepared with the same buffer as the plant extract control) at concentrations of 3 mg/kg SalE1a, or 3 mg/kg SalE1a, 1 mg/kg SalE1b, 1 mg/kg SalE2, 1 mg/kg SalE7 or 0.3 mg/kg SalE1a, 0.1 mg/kg SalE1b, 0.1 mg/kg SalE2, and 0.1 mg/kg SalE7. Treated meat trims were further incubated at room temperature for 30 min. Aliquots of meat trims corresponding to ~40 g were packed into BagFilter^®^400 P sterile bags (Interscience) and stored for 1 h, 1 d and 3 d at 10 °C, which represents realistic industrial meat processing conditions that are permissive but suboptimal for bacterial growth.

In total, meat samples were incubated at room temperature for 1.5 h during salmocin treatment before they were sealed and stored at 10 °C. For analysis of bacterial populations, poultry aliquots were homogenized with 4 vol. peptone water using Bag Mixer^®^400CC^®^ homogenizer (settings: gap 0, time 30 s, speed 4; Interscience) and colony forming units (CFU) of *S. enterica* were enumerated on XLD medium (Sifin Diagnostics) supplemented with 25 µg/ml nalidixic acid upon plating of serial dilutions of microbial suspensions. Samples were analysed in quadruplicate.

### Statistical analysis of data

The efficacy of the salmocin treatment in reducing the number of viable pathogenic *Salmonella* in the experimentally contaminated meat samples was evaluated by comparing the data obtained with the carrier-treated control samples and salmocin-treated samples by two-tailed unpaired parametric t-test with 6 degrees of freedom using GraphPad Prism v. 6.01.

### Simulated gastro-duodenal digestion *in vitro*

Gastric (phase I) and duodenal (phase II) digestion *in vitro* was performed with plant-produced lyophilized purified SalE7, SalE1a or SalE1b dissolved in Millipore water by the method described previously^10^. Briefly, individual salmocins were incubated in simulated gastric fluid (SGF) and simulated intestinal fluid (SIF) containing pepsin or trypsin and chymotrypsin in physiological concentrations, respectively. Incubation with pepsin in SGF for up to 60 min was followed by incubation with trypsin and chymotrypsin in SIF for up to 3 h. Aliquots of the reactions were evaluated for antimicrobial activity and protein degradation pattern by SDS-PAGE and Coomassie-staining upon different intervals of incubation. For SDS-PAGE analysis, pre-cast 4-20% Mini-PROTEAN^®^ TGX™ gels (Bio-Rad Laboratories) with loading corresponding to 1.5 µg SalE1a and SalE7 or 1 µg SalE1b proteins per lane were used.

### Matrix-assisted laser desorption/ionization (MALDI) time-of-flight (TOF) mass spectrometry (MS)

For proteolytic digestion, TSP extracts prepared from plant material expressing salmocins with 5 vol. 20 mM Na citrate, 20 mM NaH_2_PO_4_, 30 mM NaCl, pH 5.5 were subjected to SDS-PAGE and Coomassie-stained SDS gel bands containing 5 µg of protein were excised and destained by consecutive washing with 100 mM NH_4_HCO_3_ and 100 mM NH_4_HCO_3_ in acetonitrile (ACN)/H_2_O (50; 50, v/v). Disulfide bonds were reduced with 10 mM DTT for 45 min at 50 °C followed by alkylation with 10 mg/ml of iodoacetamide for 60 min. Destained and alkylated gel bands were then subjected to proteolytic digestion with different sequencing grade endoproteinases (Promega, Madison, USA). Protease:protein ratio in the digestion solutions was adjusted to 1:20 (w/w) and digestions were carried out for 12 h at 25 °C (chymotrypsin) or 37 °C (Asp-N, Glu-C, Lys-C, trypsin). Proteolytic peptides were extracted by consecutive washing with H_2_O, ACN/H_2_O/trifluoroacetic acid (50; 45; 5, v/v/v) and ACN, respectively. Extraction solutions were combined, concentrated in a vacuum centrifuge and resolubilized in H_2_O/acetic acid (90; 10, v/v).

Proteolytic salmocin peptides obtained as described above or purified intact plant-produced salmocin SalE1a, SalE1b and SalE7 proteins were purified for mass spectrometry by solid-phase extraction using C4 or C18 bonded silica material (ZipTip^®^, Millipore, Darmstadt, Germany) and elution solutions were co-crystallized on a MALDI ground steel target with 2,5-dihydroxyacetophenone as well as 2,5-dihydroxybenzoic acid matrix (Bruker Daltonics, Bremen, Germany).

Mass spectra were acquired on a MALDI-TOF/TOF mass spectrometer (Autoflex SpeedTM, Bruker Daltonics, Bremen, Germany) with positive polarity in linear mode for molecular mass determination and in reflector mode for protein sequencing by In-source decay (ISD) analysis. The matrix crystals were irradiated with a Nd:YAG laser (Smart beam-IITM, Bruker Daltonics, Bremen, Germany) at an emission wavelength of 355 nm and set to a pulse rate of 1 kHz.

MS and MS/MS spectra were recorded with flexControl (version 3.4, Bruker Daltonics, Bremen, Germany) by accumulation of at least 5000 or 10000 laser shots (per sample spot), respectively. Laser energy was set slightly above the threshold for MS experiments and set to maximum for MS/MS analyses. Spectra processing was carried out with flexAnalysis (version 3.4, Bruker Daltonics, Bremen, Germany) by applying baseline subtraction with TopHat algorithm, smoothing with Savitzky-Golay algorithm and peak detection with SNAP algorithm.

The mass spectrometer was calibrated using a set of standard peptides and proteins with known masses (Peptide Calibration Standard II, Protein Calibration Standard I and II, Bruker Daltonics, Bremen, Germany).

Determination of the intact molecular mass was based on the mass-to-charge-ratios (*m/z*) of single and multiple charged molecular ions.

Sequencing of protein termini was carried out by ISD analysis. The annotation of ISD fragment spectra was carried using BioTools (version 3.2, Bruker Daltonics, Bremen, Germany) by in silico generation of *m/z* values for fragment ions and their comparison with the *m/z* values of the fragment signals observed within the acquired ISD spectra. This approach enabled the identification of the terminal amino acid sequences as well as of present modifications.

For protein sequencing analysis, only fragment (MS/MS) spectra were used for the identification of proteolytic peptides and the annotation was carried out with PEAKS Studio (version 7.5, Bioinformatics Solutions Inc., Waterloo, Canada). Identification of proteins and verification of their amino acid sequences was performed by searching the MS/MS data against the NCBI nr database and the UniProt/SwissProt database to which the sequences of the salmocins were appended, respectively. Database search was performed with a parent mass error tolerance of 50 ppm and a fragment mass error tolerance of 0.5 Da. The maximum number for both missed cleavages as well as post-translational modifications for one proteolytic fragment was set to 3. Non-specific cleavage was allowed for both protein termini.

### Data availability

The authors declare that all data supporting the findings of this study are available within the article and supplementary information.

## Electronic supplementary material

Supplementary Information
