# Supplementary material for: Plant-made Salmonella bacteriocins salmocins for control of Salmonella pathovars
Source: Sci Rep. 2018 Mar 6;8:4078. doi: 10.1038/s41598-018-22465-9 (PMC5840360; doi:10.1038/s41598-018-22465-9)
Supplement: Supplementary file 1 — Supplementary Information [file 41598_2018_22465_MOESM1_ESM.pdf]

Plant-made *Salmonella* bacteriocins salmocins for control of *Salmonella* pathovars

Tobias Schneider[1,+], Simone Hahn-Löbmann[1,+], Anett Stephan[1], Steve Schulz[1], Anatoli Giritch[1,*], Marcel Naumann[2], Martin Kleinschmidt[2], Daniel Tusé[3], and Yuri Gleba[1]

[1]Nomad Bioscience GmbH, Biozentrum Halle, Weinbergweg 22, D-06120 Halle (Saale), Germany

[2]Fraunhofer Institute for Cell Therapy and Immunology, Department of Drug Design and Target Validation, Biozentrum Halle, Weinbergweg 22, D-06120 Halle (Saale), Germany

[3]DT/Consulting Group, 2695 13th Street, Sacramento, CA 95818, USA

[+]these authors contributed equally to this work.

[*]Correspondence should be addressed to A.G. (giritch@nomadbioscience.com)

Supplementary Information

Supplementary Tables

Supplementary Table 1. List of salmocin immunity proteins used in the study. Salmocin specificities and accession numbers are presented.

No.	Immunity protein	Specificity	GenBank Accession No.
1	SImmE2	SalE2 (DNase)	KTM78571.1
2	SImmE7	SalE7 (DNase)	KSU39546.1

Supplementary Table 2. List of *Salmonella enterica* ssp. *enterica* strains analysed for antimicrobial susceptibility. Serotype antigenic formula is given in (Subspecies [space] O antigens [colon] Phase 1 H antigens [colon] Phase 2 H antigens) as provided by the supplier. Numbers in source of supply correspond to 1 - Microbiologics, Inc. (St. Cloud, USA), 2 – LGC Standards (Teddington, UK), 3 – Robert Koch Institute, national reference centre for salmonellosis and other enteric pathogens (Wernigerode, Germany), 4 – Leibnitz Institute DSMZ - German Collection of Microorganisms and Cell Cultures (Braunschweig, Germany), 5 - National Collection of Type Cultures (Salisbury, UK). Strains marked with “ were used for antmicrobial susceptibility testing in triplicate experiments. [†]The number of incidences refers to laboratory-confirmed human *Salmonella* infections (US) reported to CDC 2003-2012 published in National Enteric Disease Surveillance: *Salmonella* Annual Report, 2013 (CDC, June 2016; https://www.cdc.gov/nationalsurveillance/pdfs/salmonella-annual-report-2013-508c.pdf).

No.	culture collection reference No.	serotype	serotype antigenic formula	source of supply	No. of incidences[†]
1“	ATCC® 13076™*	Enteritidis	I 1,9,12:g,m:-	1	74450
2“	ATCC® 49223™*		I 9,12:g,m	1	
3“	ATCC® 14028™*	Typhimurium	I 4,5,12:i:1,2	1	70251
4“	ATCC® 13311™*		I 4,5,12:i:1,2	1	
5“	ATCC® 6962™*	Newport	I 6,8:e,h:1,2	1	44675
6“	ATCC® 10721™*	Javiana	I 1,9,12:l,z28:1,5	2	22868
7“	ATCC® BAA-1593™		I 9,12:-:1,5	2	
8“	ATCC® 8326™*	Heidelberg	I 4,5,12:r:1,2	1	15912
9	17-00918	-	I 4,[5],12:i:-	3	13567
10“	ATCC® 8387™*	Montevideo	I 6,7:g,m,s:-	2	11377
11“	ATCC® 8388™*	Muenchen	I 6,8:d:1,2	2	9589
12“	ATCC® 9712™*	Saintpaul	I 1,4,5,12:e,h:1,2	2	9420
13“	ATCC® BAA-1675™	Infantis		2	8106
14“	ATCC® 9239™*	Oranienburg	I 6,7:m,t:-	2	7514
15“	ATCC® 700136™*	Braenderup	I 6,7:e,h:e,n,z15	2	7371
16“	ATCC® BAA-2739™	Mississippi	I 13,23:b:1,5	2	5693
17“	ATCC® 8391™*	Thompson	I 6,7:k:1,5	2	5660
18“	ATCC® 51957™*	Agona	I 4,12:f,g,s:-	1	5072
19	16-04932	Paratyphi B var. L(+) tartrate +	I 4,5:b:1,2	3	4624
20“	ATCC® 9115™*	Bareilly	I 6,7:y:1,5	2	3704
21“	NCTC 4840	Poona	I 13,22:z:1	1	2977
22	16-4909	Hadar	I 6,8:z10:e,n,x	3	2857
23	16-05099	Schwarzengrund	I 4:d:1,7	3	2835

No.	culture collection reference No.	serotype	serotype antigenic formula	source of supply	No. of incidences†
24“	ATCC® 8392™*	Berta	I 9,12:f,g,t:-	2	2779
25“	ATCC® 9270™*	Anatum	I 3,10:e,h:1,6	1	2753
26	16-04966	Stanley	I 4,5:d:1,2	3	2438
27	15-04731	Litchfield	I 6,8:e,v:1,2	3	2386
28	10-03610	Hartfort	I 6,7:y:e,n,x	3	2312
29“	ATCC® 51958™*	Mbandaka	I 6,7:z10:e,n,z15	2	2286
30	16-03044	Panama	I 9:e, v:1,5	3	1903
31	16-04172	-	I 4,[5],12:b:-	3	1860
32	14-03918	Sandiego	I 4,5:e,n:e,n,z15	3	1759
33“	ATCC® 9150™*	Paratyphi A	I 1,2,12:a:-	1	1731
34“	DSM 10062	Senftenberg	I 1,3,19:g,s,t:-	4	1594
35	NCTC 7077	Norwich	I 6,7:e, h:1,6	5	1481
36	16-05141	Tennessee	I 6,7:z29:-	3	1476
37	16-05288	Rubislaw	I 11:r:e,n,x	3	1394
38“	ATCC® 6960™*	Derby	I 1,4,12:f,g:-	2	1392
39	07-06267	-	I 13,23:b:-	3	1275
40	16-05246	Give	I 3,10:l,v:1,7	3	1250
41	16-05252	Paratyphi B	I 4,5:b:1,2	3	1249
42	14-04905	Miami	I 9:a:1,5	3	1087
43“	ATCC® 15480™*	Dublin	I 1,9,12:g,p:-	2	1086
44“	ATCC® 9263™*	Kentucky	I (8),20:i:z6	2	984
45	16-05080	Brandenburg	I 4:l,v:e,n,z15	3	963
46	16-04827	Virchow	I 6,7:r:1,2	3	961
47	16-02846	Gaminara	I 16:d:1,7	3	953
48	17-00031	Weltevreden	I 3,10:r:z6	3	876
49	16-05006	Bovismorbisficans	I 6,8:r:1,5	3	839
50	17-00039	Manhattan	I 6,8:d:1,5	3	836
51	14-05486	Adelaide	I 35:f,g:-	3	820
52	16-05394	Uganda	I 3,10:e,z13:1,5	3	817
53	15-03669	Pomona	I 28:Y:1,7	3	781
54	16-04580	Muenster	I 3,10:e,h:1,5	3	756
55	15-01597	Kiambu	I 4:z:1,5	3	699
56	15-02141	Blockley	I 6,8:k:1,5	3	688
57	16-04687	Ohio	I 6,7:b:e,w	3	656
58	16-05313	Hvittingfoss		3	620
59	16-01351	Reading	I 4,5:e,h:1,5	3	619
60	11-00574	Inverness	I 38:k:1,6	3	587
61	13-02698	Urbana	I 30:b:e,n,x	3	565
62	16-05172	London	I 3,10:e,v:1,6	3	480
63	14-05710	Johannesburg	I 40:b:e,n,x	3	443
64	16-05303	Chester		3	435
65	16-02928	Havana	I 13,23:f,g:-	3	395
66	16-01712	Bredeney	I 4:l,v:1,7	3	383

No.	culture collection reference No.	serotype	serotype antigenic formula	source of supply	No. of incidences[†]
67	15-01962	-	I6,7:-:1,5	3	366
68	15-02251	Telelkebir	I 13,23:d:e,n,z15	3	361
69“	ATCC® 10723™*	Cerro	I 18:z4,z23:-	2	346
70	16-04988	Albany	I 8,20:z4:z24	3	344
71	16-02205	Agbeni	I 13,23:g,m:-	3	343
72	14-02295	Minnesota	I 21:b:e,n,x	3	337
73	14-01914	Worthington	I 13,23:z:e,w	3	336
74	16-05041	Rissen	I 6,7:f,g:-	3	312
75	16-02392	Oslo	I 6,7:a:e,n,x	3	306
76	11-06323	Baildon	I 9,46:a:e,n,x	3	278
77	16-02147	Cotham	I 28:i:1,5	3	253
78	15-03689	Ealing	I 35:g,m,s	3	237
79	418	Lomalinda	I 9, 12:a:e, n, x	3	232
80	15-01471	Cubana	I 13,23:z29	3	213
81	09-01912	Carrau	I 6,14,24:y:1,7	3	209
82	16-02464	Eastbourne	I 9:e,h:1,5	3	203
83	17-00172	Monschaui	I 35:m,t:-	3	201
84	15-01577	Alachua	I 35:z4,z23:-	3	193
85	16-03390	Corvallis	I 8,20:z4, z23	3	189
86	16-00455	Potsdam	I 6,7:e,v:e,n,z15	3	187
87	17-00107	Meleagridis	I 3,10:e,n:e,w	3	169
88	16-05286	Indiana		3	158
89	15-02982	Concord	I 6,7:l,v:1,2	3	157
90	03-08607	-	I 6,7:k:-	3	149
91“	ATCC® 10708™*	Cholerasius	I 6,7:C:1,5	1	148
92	16-03583	Altona	I 8,20:r:z6	3	145
93	11-07920	Pensacola	I 9:m,t:-	3	143
94	01-02501	Othmarschen	I 6,7:g,m:-	3	134
95	12-02378	-	I 4,[5],12:-:1,2	3	130
96	16-05338	Lovingstone	I 6,7:d:e,w	3	123
97	15-03273	Grumpensis	I 13,23:d:1,7	3	122
98	15-04797	Wandsworth	I 39:b:1,2	3	118
99	13-04865	Kintambo	I 13,23:m,t:-	3	114
100	13-05516	Edinburgh		3	113
101	16-04965	Kottbus	I 6,8:e,h:1,5	3	109
102	15-00740	Durban	I 9:a:e,n,z15	3	104
103“	NCTC 6017	Abony	I 4,12,27:b:e,n,x	1	60
104“	ATCC® 9842™*	Bispebjerg	I 4,12:a:enx	1	1
105“	ATCC® 15611™*	Vellore	I 1,4,12,27:z10:z35	1	-
106“	ATCC® 13036™*	Pullorum	I 9,12:-:-	1	-
107	ATCC® 12002™*	Tallahassee	I 6,8:z4,z32:-	1	67
108“	DSM 4883	Gallinarum	I 9:-:-	4	-
109“	DSM 13674		I 9,12:-:-	4	

Supplementary Table 3. List of *E. coli* bacteriocins (colicins) used in the study. Cytotoxic activities and accession numbers are presented.

No.	Colicin	Activity	Accession No.
1	colS4	pore-forming	CAB46008.1
2	col5	pore-forming	CAA61102.1
3	col10	pore-forming	CAA57998.1
4	colIa	pore-forming	WP_001283344.1
5	colIb	pore-forming	AAA23188.1
6	colM	cell wall-inhibition	AAA23589.1

Supplementary Table 4. List of *E. coli* STEC strains used in this study.

No.	Culture collection reference #	Serotype	Characteristics	Source of supply
1	CDC 03-3014	O26:H11	Positive for virulence genes *stx1* and/or *stx2* and *eae*	Big 7 STEC QC Set (#5219, Microbiologics Inc., St. Cloud, Minnesota USA)
2	CDC 00-3039	O45:H2		
3	CDC 06-3008	O103:H11		
4	CDC 2010C-3114	O111:H8		
5	CDC 02-3211	O121:H19		
6	CDC 99-3311	O145:NM		
7	ATCC® 35150™	O157:H7		

Supplementary Table 5. Identity and integrity studies on plant-produced salmocins. MALDI-TOF/TOF mass spectrometry analysis of salmocin-containing TSP extracts of *N. benthamiana* or purified salmocins by peptide mass fingerprinting or by sequencing of protein termini by in-source decay and molecular mass determination, respectively. Proteases used for generation of peptide fragments are indicated. The identity of salmocins was confirmed by searching MS/MS datasets obtained against NCBI non-redundant database. Obtained molecular masses indicate that the proteins were intact. ND, not detected; PTM post-translational modification; aa, amino acid sequence.

salmocin-containing TSP extracts of *N. benthamiana*						
No.	salmocin	peptide mass fingerprinting				
		Peptides annotated	Amino acid coverage (trypsin, Asp-N, chymotrypsin, Glu-C, Lys-C, combined)	Proteoform	N-terminus (aa and PTM)	C-terminus (aa and PTM)
1	SalE2	44	56,3%	1<br>2	H_2N-SGGDGIGHNS[…]<br>(M cleaved, no PTM)<br>Acetyl-SGGDGIGHNS[…]<br>(M cleaved, acetylation)	[…]KLHIDIHRGK-OH<br>(intact, no PTM)
2	SalE7	31	33,3%	-	ND	[…]KRHIDIHRGQ-OH<br>(intact, no PTM)
3	SalE3	18	31,5%	-	ND	ND
4	SalE1a	14	34,2%	1	Acetyl-ADNTIAYYED[…]<br>(M cleaved, acetylation)	ND
5	SalE1b	25	47,1%	-	ND	ND

salmocins purified from *N. benthamiana*							
No.	salmocin	Batch	Theoretical mass of intact protein (Da)	Molecular mass		in-source decay	
				Average mass (Da) (PTM)	Proteoform	N-terminus (aa and PTM)	C-terminus (aa and PTM)
2	SalE7	1<br>2<br>3	62259.4	62111.0<br>62126.9<br>62137.8<br>(N-terminus: M cleaved)	1<br>1<br>1	H_2N-SGGDGG[…]<br>(M cleaved, no PTM)<br>H_2N-SGGDGG[…]<br>(M cleaved, no PTM)<br>ND	[…]KRHIDIHRGQ-OH<br>(intact, no PTM)<br>[…]KRHIDIHRGQ-OH<br>(intact, no PTM)<br>ND
4	SalE1a	1<br>2<br>3	52811.3	ND<br>ND<br>52722.1<br>(N-terminus: M cleaved, acetylation)	1<br>1<br>1	ND<br>ND<br>ND	ND<br>ND<br>ND
5	SalE1b	1<br>2<br>3	57583.1	57486.3<br>57470.0<br>57480.7<br>(N-terminus: M cleaved)	1<br>1<br>1	ND<br>ND<br>ND	ND<br>ND<br>ND

Supplementary Figures

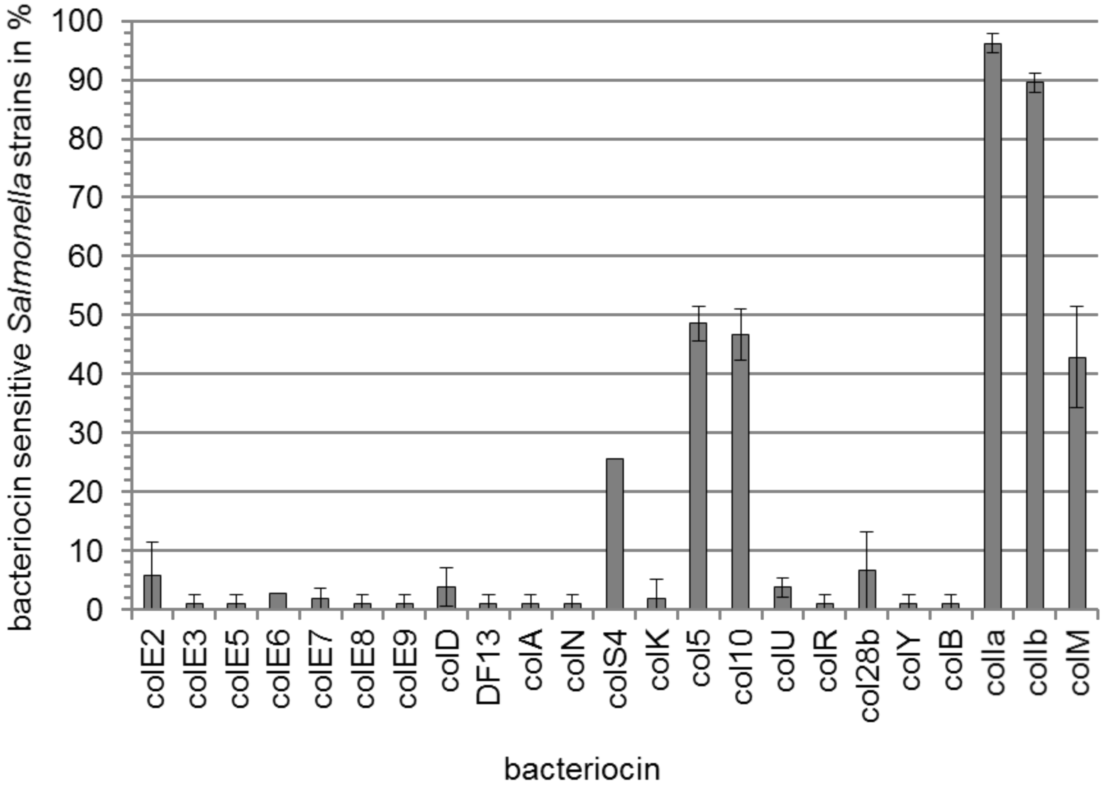

Supplementary Figure 1. Activity spectrum of bacteriocins, most of them colicins from *E. coli*, against *Salmonella enterica* ssp. *enterica* serotypes. Semi-quantitative evaluation of the specific antimicrobial activity of bacteriocin-containing plant total soluble extracts by radial diffusion assay via spot-on-lawn-method of colicin-containing plant extracts against 35 *S. enterica* ssp. *enterica* strains listed in **Supplementary Table 2**. Average and standard deviation of N=3 independent experiments for the percentage of bacteriocin-sensitive strains.

a

Query: SalE2 (KTM78572.1, 583 aa) DNase
Sbjct: colE2 (AAA23068.1, 581 aa) DNase

```
Score          Expect   Method                        Identities     Positives      Gaps
729 bits(1882) 0.0      Compositional matrix adjust.  398/586(68%)   455/586(77%)   8/586(1%)
Query  1    MSGGDGIGHNSGAHSTGG-VNGSSSGRGGSSSGGGNNPNSGPGWGTTHTPDGHDIHNYNP  59
            MSGGDG GHN+GAHST G +NG  +G G          + G GW + + P G    +
Sbjct  1    MSGGDGRGHNTGAHSTSGNINGGPTGLGVGGGA-----SDGSGWSSENNPWGGGSGSGIH  55
Query  60   GEFGGGGHKPGGNGGNHSGGTGDGQPPGAA--MAFGFPALVPAGAGGLAVTVSGDALAAA  117
               G G    GGNG +  G    G     A  +AFGFPAL   GAGGLAV++S  AL+AA
Sbjct  56   WGGGSGHGNGGGNGNSGGGSGTGGNLSAVAAPVAFGFPALSTPGAGGLAVSISAGALSAA  115
Query  118  IADVLAVLKGPFKFGAWGIALYGILPTEIAKDDPRMMSKIVTSLPADAVTESPVSSLPLD  177
            IAD++A LKGPFKFG WG+ALYG+LP++IAKDDP MMSKIVTSLPAD +TESPVSSLPLD
Sbjct  116  IADIMAALKGPFKFGLWGVALYGVLPSQIAKDDPNMMSKIVTSLPADDITESPVSSLPLD  175
Query  178  QATVSVTKRVTDVVKDERQHIAVVAGVPASIPVVDAKPTTHPGVFSVSVPGLPDLQVSTV  237
            +ATV+V  RV D VKDERQ+I+VV+GVP S+PVVDAKPT  PGVF+ S+PG P L +S
Sbjct  176  KATVNVNVRVVDDVKDERQNISVVSGVPMSVPVVDAKPTERPGVFTASIPGAPVLNISVN  235
Query  238  KNAPAMTALPRGVTDEKDRTVHPAGFTFGGSSHEAVIRFPKESGQAPVYVSVTDVLTPEQ  297
             + P +  L  GVT+  D+ V PAGFT GG++ +AVIRFPK+SG   VYVSV+DVL+P+Q
Sbjct  236  NSTPEVQTLSPGVTNNTDKDVRPAGFTQGGNTRDAVIRFPKDSGHNAVYVSVSDVLSPDQ  295
Query  298  VKQRQDEENRRQQEWDATHPVEVAERNYRLASDELNRANVDVAGKQERQIQAAQAVAARK  357
            VKQRQDEENRRQQEWDATHPVE AERNY  A  ELN+AN DVA  QERQ +A Q   +RK
Sbjct  296  VKQRQDEENRRQQEWDATHPVEAAERNYERARAELNQANEDVARNQERQAKAVQVYNSRK  355
Query  358  GELDAANKTFADAKEEIKKFERFAHDPMAGGHRMWQMAGLKAQRAQNEVNQKQAEFNAAE  417
             ELDAANKT ADA  EIK+F RFAHDPMAGGHRMWQMAGLKAQRAQ +VN KQA F+AA
Sbjct  356  SELDAANKTLADAIAEIKQFNRFAHDPMAGGHRMWQMAGLKAQRAQTDVNNKQAAFDAAA  415
Query  418  KEKADADAALNVALESRKQKEQKAKDASDKLDKENKRNHPGKATGKGQPVGDKWLEDAGK  477
            KEK+DADAAL+ A E RKQKE K KDA DKLDKE+KRN PGKATGKG+PVGDKWL+DAGK
Sbjct  416  KEKSDADAALSAAQERRKQKENKEKDAKDKLDKESKRNKPGKATGKGKPVGDKWLDDAGK  475
Query  478  EAGAPVPDRIADKLRDKEFKNFDDFRKKFWEEVSKDPELSKQFIPGNKKRMSQGLAPRAR  537
            ++GAP+PDRIADKLRDKEFKNFDDFRKKFWEEVSKDP+LSKQF   NK  + +G AP AR
Sbjct  476  DSGAPIPDRIADKLRDKEFKNFDDFRKKFWEEVSKDPDLSKQFKGSNKTNIQKGKAPFAR  535
Query  538  NKDTVGGRRSFELHHDKPISQDGGVYDMDNIRVTTPKLHIDIHRGK  583
             KD VGGR  FELHHDKPISQDGGVYDM+NIRVTTPK HIDIHRGK
Sbjct  536  KKDQVGGRERFELHHDKPISQDGGVYDMNNIRVTTPKRHIDIHRGK  581
```

b

Domain, potential function	Domain, accession	Interval SalE2	Interval colE2	Identities	Positives	Gaps	Coverage
Trans-location	pfam03515	aa 88-313	aa 33-311	155/224 (69%)	186/224 (83%)	0/227 (0%)	80%
Receptor binding	pfam11570	aa 316-449	aa 314-447	98/134 (73%)	106/134 (79%)	0/134 (0%)	100%
Toxic activity	cd00085	aa 543-580	aa 541-578	34/38 (89%)	35/38 (92%)	0/38 (0%)	100%

Supplementary Figure 2. (*a*) Alignment of salmocin E2 (SalE2) from *Salmonella* and colicin E2 (colE2) from *E. coli*. Protein sequences retrieved from GenBank were compared using BLASTP 2.6.1+ program[1]. Detected conserved domains are marked with different colors and similarities are given in (*b*).

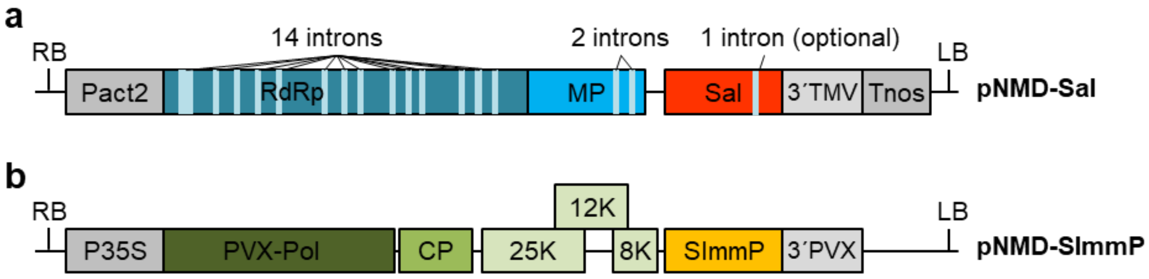

c

No.	Salmocin	Construct		Position of intron in salmocin
		Name	Internal name	
1	SalE2	pNMD-SalE2	pNMD28161	V510
2	SalE3	pNMD-SalE3	pNMD28151	G554
3	SalE7	pNMD-SalE7	pNMD28172	V532
4	SalE1a	pNMD-SalE1a	pNMD28191	-
5	SalE1b	pNMD-SalE1b	pNMD28204	-

No.	Immunity protein	Construct	
		Name	Internal name
1	SImmE2	pNMD-SImmE2	pNMD28222
2	SImmE7	pNMD-SImmE7	pNMD28232

Supplementary Figure 3. Schematic representation of T-DNA regions of plasmid constructs used for transient *Agrobacterium*-mediated expression of salmocins. (*a*) salmocin-expressing TMV-based vector capable of cell-to-cell movement (pNMD-Sal). (*b*) PVX-based vector capable of systemic movement for the expression of salmocin immunity proteins (pNMD-SImmP). LB and RB, binary left and right borders, respectively; Pact2, *Arabidopsis thaliana* actin 2 promoter; Tnos, *nos* terminator; RdRp, RNA-dependent RNA polymerase of TVCV (Turnip Vein-Clearing Virus); MP, movement protein; 3´TMV, 3´untranslated region of TMV; P35S, *CaMV* 35S promoter; PVX-Pol, PVX RdRp; CP, PVX coat protein; 25K, 12K, 8K, PVX triple gene block; 3´PVX, 3´CP coding sequence and 3´untranslated region of PVX. Introns inserted in RdRp and MP sequences of TMV-based vectors were previously described in Marillonnet et al.[2]. (*v*) List of plasmid constructs used in this study. Position of intron in salmocin refers to amino acid codon in which the intron (from *Ricinus communis cat 1* gene for catalase CAT1 (GenBank #D21161.1, base pairs 679-867)) was inserted and position of encoded amino acid in the sequence.

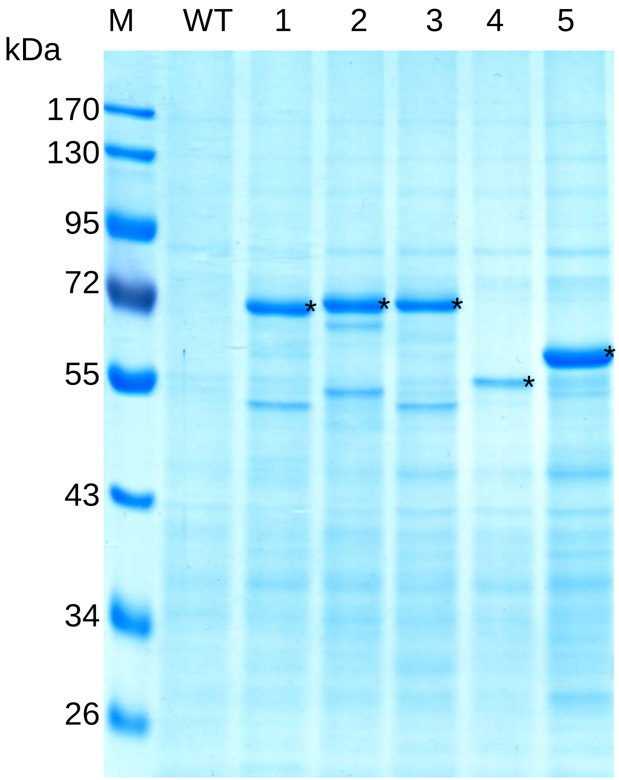

Supplementary Figure 4. Soluble selective extraction of salmocins from *N. benthamiana* plant material upon transient expression. Leaf material was vacuum-infiltrated with agrobacteria carrying TMV or TMV and PVX vectors. Coomassie-stained SDS protein gel loaded with TSP extracts corresponding to 3 mg fresh weight plant material, prepared with 20 mM citric acid pH 4.0, 20 mM NaH_2PO_4, 30 mM NaCl. Plant material was harvested 5 dpi (days post infiltration) for SalE1b and 6 dpi for SalE3, SalE7 and SalE1a. All constructs used are described in **Supplementary Fig. 3**. Analyzed extracts were prepared from plant material expressing SalE2 (lane 1), SalE3 (lane 2), SalE7 (lane 3), SalE1a (lane 4) and SalE1b (lane 5) or from (WT) non-transfected leaf tissue. SalE2 and SalE7 were co-expressed with their respective immunity proteins. Asterisks mark recombinant proteins.

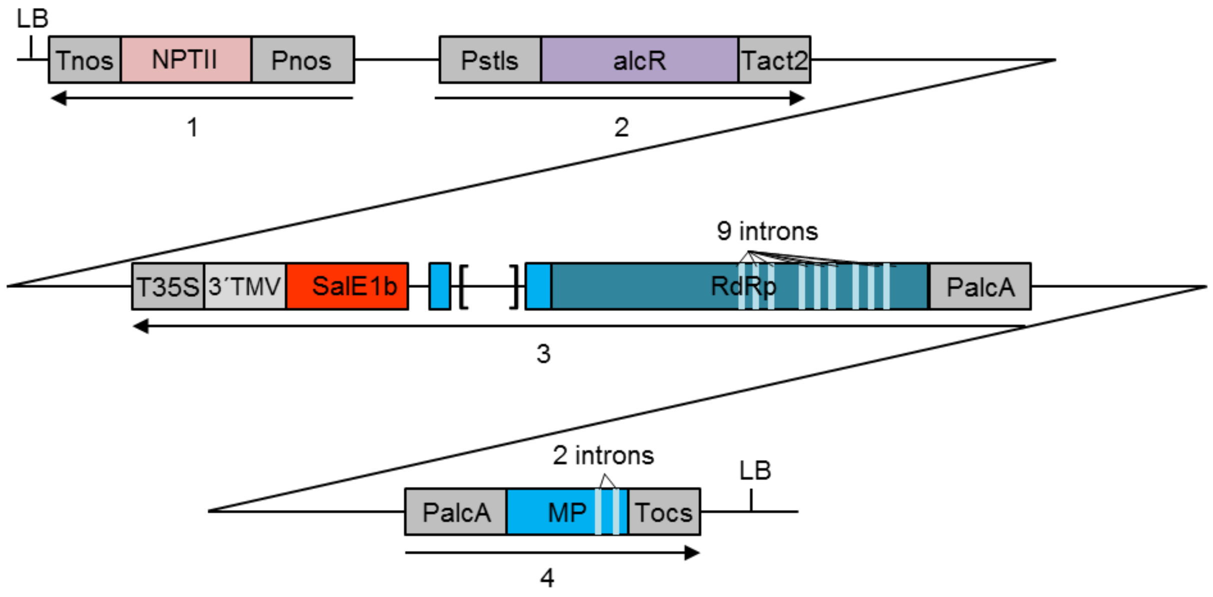

Supplementary Figure 5. Schematic representation of T-DNA regions of SalE1b-encoding plasmid construct (pNMD35541) used for stable plant transformation. The T-DNA region is composed of 4 expression cassettes for: 1) constitutive expression of kanamycine resistance transgenic plant selection marker, 2) constitutive expression of alcR transcriptional activator, 3) ethanol-inducible expression of salmocin SalE1b and 4) ethanol-inducible expression of TMV MP. Arrows indicate orientation of expression cassettes. For tight control of viral replicon activation in non-induced state, the viral vector is deconstructed in the 2 components, replicon and MP (expression cassettes 3 and 4)[3]. LB and RB, binary left and right borders, respectively; Tnos and Pnos, terminator and promoter of the *Agrobacterium* nopaline synthase gene; NPTII, neomycin phosphotransferase II; Pstls, promoter of potato *ST-LS1* gene; alcR, *Aspergillus nidulans alcR* ORF; Tact2, *Arabidopsis thaliana* actin 2 terminator; T35S, *CaMV* 35S terminator; 3´TMV, 3´untranslated region of TMV; RdRp, RNA-dependent RNA polymerase; pAlcA, *Aspergillus nidulans* alcohol dehydrogenase (*alcA*) promoter; MP, movement protein; Tocs terminator of *Agrobacterium* octopine synthase gene; [] deletion of MP.

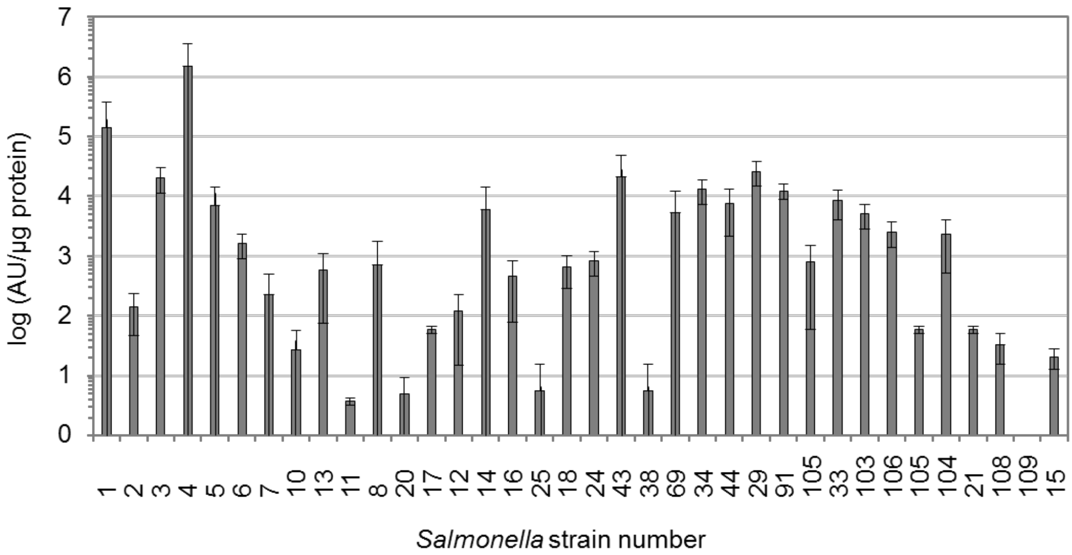

Supplementary Figure 6. Specific activity of individual salmocin SalE2 against *Salmonella enterica* ssp. *enterica* serovars. Semi-quantitative evaluation of the average specific antimicrobial activity of salmocin SalE2-containing plant total soluble extracts against 36 *S. enterica* ssp. *enterica* strains listed in **Supplementary Table 2**. The antimicrobial activity was tested using radial diffusion assay via spot-on-lawn-method and calculated in arbitrary units (AU) per µg recombinant protein (average of 3 independent experiments, error bars correspond to standard deviation).

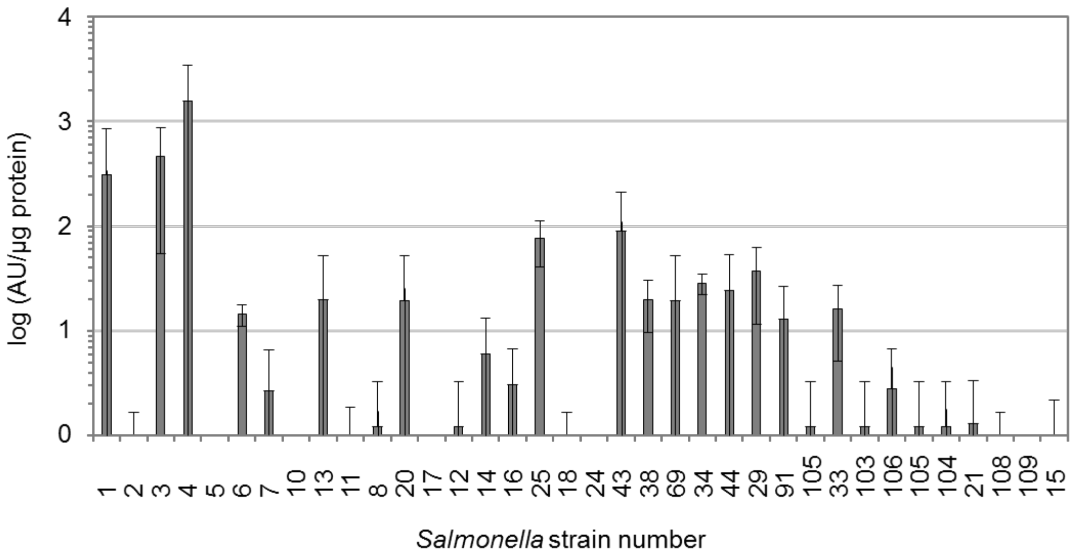

Supplementary Figure 7. Specific activity of individual salmocin SalE3 against *Salmonella enterica* ssp. *enterica* serovars. Semi-quantitative evaluation of the average specific antimicrobial activity of salmocin SalE3-containing plant total soluble extracts against 36 *S. enterica* ssp. *enterica* strains listed in **Supplementary Table 2**. The antimicrobial activity was tested using radial diffusion assay via spot-on-lawn-method and calculated in arbitrary units (AU) per µg recombinant protein (average of 3 independent experiments, error bars correspond to standard deviation).

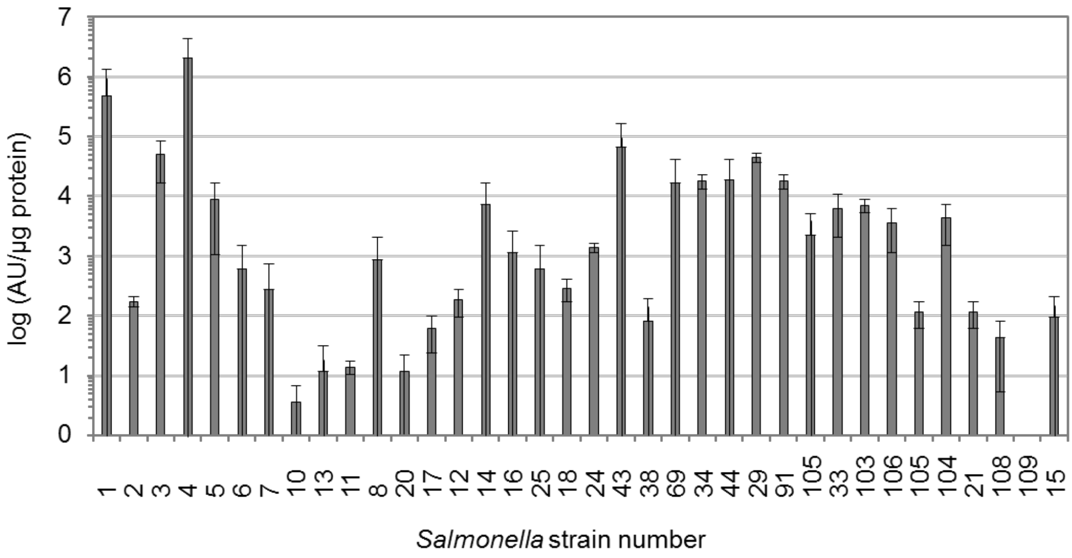

Supplementary Figure 8. Specific activity of individual salmocin SalE7 against *Salmonella enterica* ssp. *enterica* serovars. Semi-quantitative evaluation of the average specific antimicrobial activity of salmocin SalE7-containing plant total soluble extracts against 36 *S. enterica* ssp. *enterica* strains listed in **Supplementary Table 2**. The antimicrobial activity was tested using radial diffusion assay via spot-on-lawn-method and calculated in arbitrary units (AU) per µg recombinant protein (average of 3 independent experiments, error bars correspond to standard deviation).

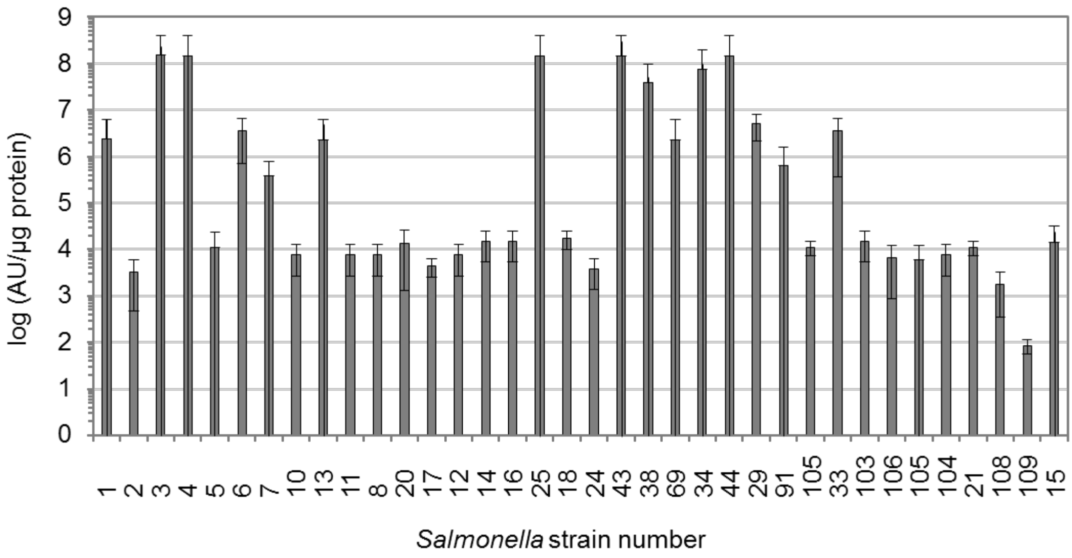

Supplementary Figure 9. Specific activity of individual salmocin SalE1a against *Salmonella enterica* ssp. *enterica* serovars. Semi-quantitative evaluation of the average specific antimicrobial activity of salmocin SalE1a-containing plant total soluble extracts against 36 *S. enterica* ssp. *enterica* strains listed in **Supplementary Table 2**. The antimicrobial activity was tested using radial diffusion assay via spot-on-lawn-method and calculated in arbitrary units (AU) per µg recombinant protein (average of 3 independent experiments, error bars correspond to standard deviation).

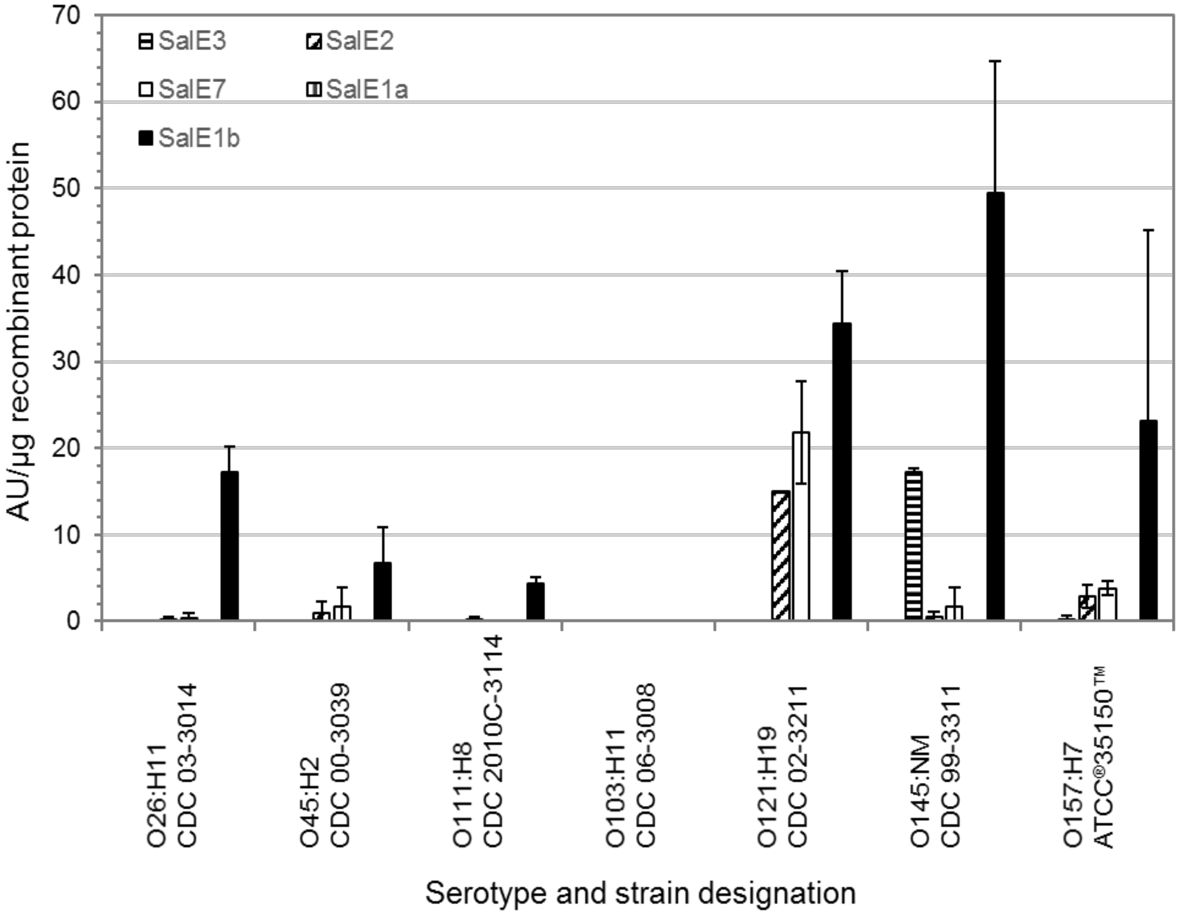

Supplementary Figure 10. Specific activity of individual salmocins against *E. coli* strains of Big7 STEC serovars. Semi-quantitative evaluation of the average specific antimicrobial activity of salmocin-containing plant total soluble extracts against 7 *E. coli* strains listed in **Supplementary Table 4**. The antimicrobial activity was tested using radial diffusion assay via spot-on-lawn-method and calculated in arbitrary units (AU) per µg recombinant protein (average and SD, N=3).

a

Salmocin	Mean percent residual antimicrobial activity						
	Not digested	Phase I – gastric digestion			Phase II – duodenal digestion		
		0 min -/- SGF	60 min -/- SGF	60 min P/- SGF	0 min P/- SIF	180 min P/- SIF	180 min P/T, C SIF
SalE1a	100%	60%	60%	0.26%	0.06%	0.06%	0.001%
SalE1b	100%	100%	100%	75%	5%	5%	0%
SalE7	100%	55%	55%	0.1%	6%	6%	0%

b

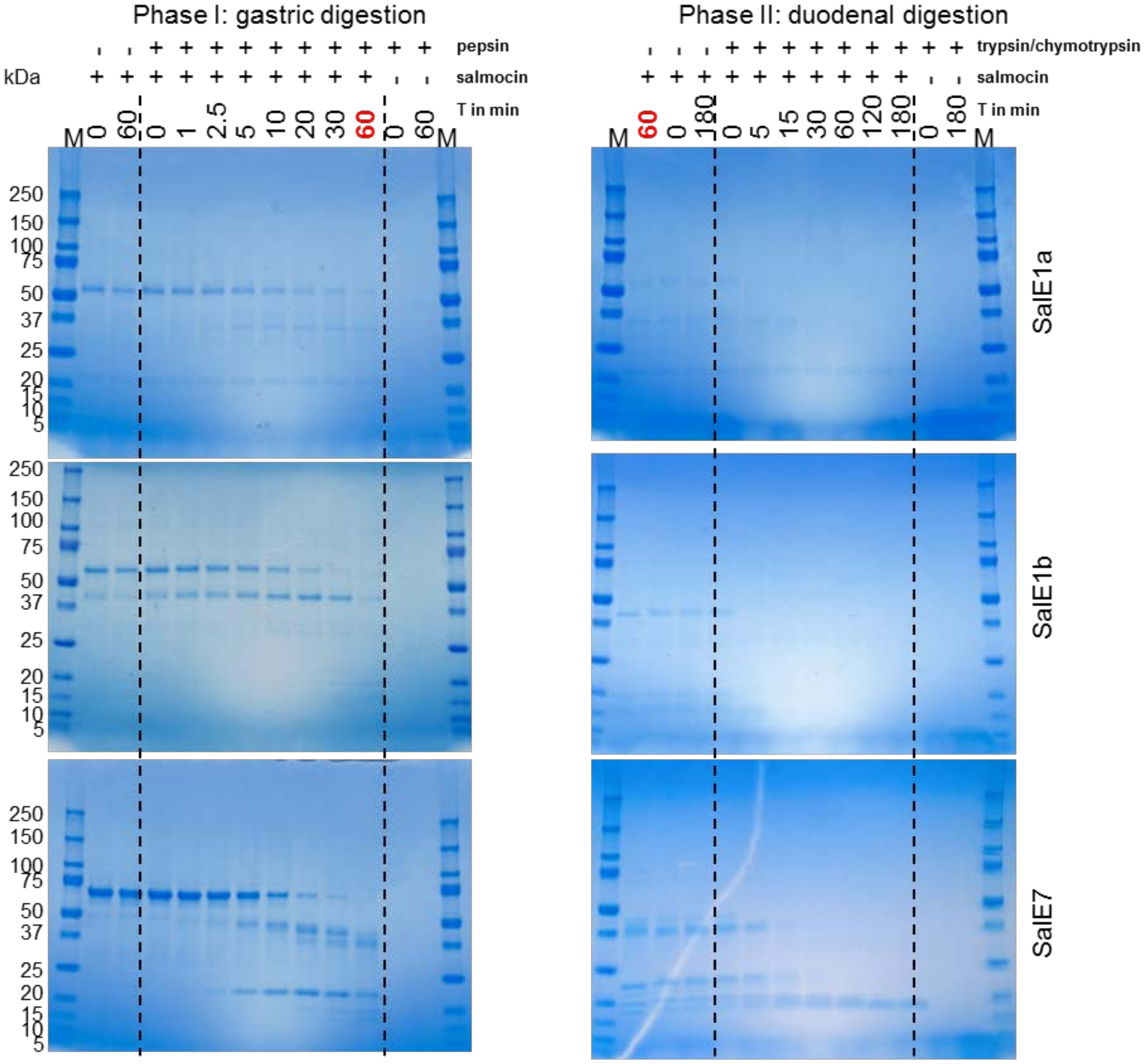

Supplementary Figure 11. *In vitro* simulated gastro-duodenal digestion of salmocins. In phase I, gastric digestion, salmocins are incubated with pepsin (P) (1:20 (w:w; pepsin:salmocin)) for up to 60 min in simulated gastric fluid (SGF, 0.15 M NaCl, pH 2.5). In phase II, duodenal digestion, salmocin peptides generated within 60 min gastric digestion are incubated for up to 180 min with trypsin and chymotrypsin (T, C) (1:4:400 (w:w:w; trypsin:chymotrypsin:salmocin)) in simulated

duodenal fluid (SIF, 4 mM sodium taurocholate, 4 mM glydeoxycholic acid, 10 mM $CaCl_2$, 25 mM Bis-Tris, pH 6.5. (*a*) Antimicrobial activies of salmocin samples during gastro-duodenal digestion relative to non-digested protein measured by analysis of serial dilutions in softagar overlay assay (*S. enterica* strain ATCC® 13076™*). (*b*) SDS-PAGE analysis of of salmocin samples during gastro-duodenal digestion, loading corresponds to 1.5 µg or 1 µg protein for SalE7 and SalE1b or SalE1a and SalE1a, respectively. Salmocin samples upon 60 min. incubation at gastric conditions (red label) were also loaded on gels analysing samples at duodenal digestion for comparison.

Supplementary References

1) Altschul, S. F. et al. Gapped BLAST and PSI-BLAST: a new generation of protein database search programs, *Nucleic Acids Res* **25**, 3389-3402 (1997).
2) Marillonnet, S., Thoeringer, C., Kandzia, R., Klimyuk, V. & Gleba, Y. Systemic *Agrobacterium tumefaciens*-mediated transfection of viral replicons for efficient transient expression in plants. *Nat Biotechnol*. **23**, 718-723 (2005).
3) Werner S., Breus, O., Symonenko, Y., Marillonnet, S. & Gleba, Y. High-level recombinant protein expression in transgenic plants by using a double-inducible viral vector. *Proc. Natl. Acad. Sci. USA* **108**,14061-14066 (2011).
